# Outcomes of 28^+1^ to 32^+0^ Weeks Gestation Babies in the State of Qatar: Finding Facility-Based Cost Effective Options for Improving the Survival of Preterm Neonates in Low Income Countries

**DOI:** 10.3390/ijerph7062526

**Published:** 2010-06-11

**Authors:** Hussain Parappil, Sajjad Rahman, Husam Salama, Hilal Al Rifai, Najeeb Kesavath Parambil, Walid El Ansari

**Affiliations:** 1 NICU Women’s Hospital, Hamad Medical Corporation, Doha, Qatar; E-Mails: drhussainparappil@gmail.com (H.P.); hsalama1@hmc.org.qa (H.S.); halrifai@hmc.org.qa (H.A.R.); knajeeb@hmc.org.qa (N.K.P.); 2 Department of Paediatrics, Weill Cornell Medical College, Doha, Qatar; 3 Faculty of Sport, Health and Social Care, University of Gloucestershire, Gloucester, UK; E-Mail: walidansari@glos.ac.uk

**Keywords:** epidemiology, gestational age, mortality, morbidity, Qatar, developing countries

## Abstract

In this retrospective study we did a comparative analysis of the outcome of 28^+1^ to 32^+0^ weeks gestation babies between the State of Qatar and some high income countries with an objective of providing an evidence base for improving the survival of preterm neonates in low income countries. Data covering a five year period (2002–2006) was ascertained on a pre-designed Performa. A comparative analysis with the most recent data from VON, NICHD, UK, France and Europe was undertaken. Qatar’s 28^+1^ to 32^+0^ weeks Prematurity Rate (9.23 per 1,000 births) was less than the UK’s (*p* < 0.0001). Of the 597 babies born at 28^+1^ to 32^+0^ weeks of gestation, 37.5% did not require any respiratory support, while 31.1% required only CPAP therapy. 80.12% of the MV and 96.28% of CPAP therapy was required for <96 hours. 86.1% of the mothers had received antenatal steroids. The 28^+1^ to 32^+0^ weeks mortality rate was 65.3/1,000 births with 30.77% deaths attributable to a range of lethal congenital and chromosomal anomalies. The survival rate increased with increasing gestational age (*p* < 0.001) and was comparable to some high income countries. The incidence of in hospital pre discharge morbidities in Qatar (CLD 2.68%, IVH Grade III 0.84%, IVH Grade IV 0.5%, Cystic PVL 0.5%) was less as compared to some high income countries except ROP ≥ Stage 3 (5.69%), which was higher in Qatar. The incidence of symptomatic PDA, NEC and severe ROP decreased with increasing gestational age (*p* < 0.05). We conclude that the mortality and in hospital pre discharge morbidity outcome of 28^+1^ to 32^+0^ weeks babies in Qatar are comparable with some high income countries. In two thirds of this group of preterm babies, the immediate postnatal respiratory distress can be effectively managed by using two facility based cost effective interventions; antenatal steroids and postnatal CPAP. This finding is very supportive to the efforts of international perinatal health care planners in designing facility-based cost effective options for low income countries.

## 1. Introduction

Very Preterm Babies (≤32 weeks of gestation) constitute 1–2% of all live births in high income countries, but account for at least one third of perinatal mortality, the majority of neonatal mortality [1–5], as well as both short-term [5] (pre discharge, in hospital) and long-term (at two years corrected age) morbidity [6–9]. Although neonatal mortality and morbidity are known to worsen with decreasing gestational age and weight at birth [1–9], the dramatic improvement in the intact survival of preterm babies has been one of the most remarkable features of neonatology in high income countries over the last three decades [9]. Correspondingly, the question of how ‘small is small’ has, over the last two decades, gradually decreased in terms of gestation period from 32 weeks to 28 weeks and then 24 weeks; and in terms of birth weight from 1,500 g to 1,000 g, to 800 g and then 500 g [10–15]. However, the long term outcomes, the cost of care of babies born ≤28 weeks (particularly ≤26 weeks) and/or birth weight ≤1,000 g (and particularly ≤750 g), and the futility of intervention at the edge of viability remains a hot debate, even among the most resource rich countries [10–15] and the care of extremely premature babies (≤28 weeks and ≤ 1,000 g at birth) is not an option for resource restricted developing countries. Instead these countries should focus on babies born ≥28 weeks gestation and ≥1,000 g at birth. Over the last decade the intact survival of this group of preterm babies has risen to a level which has put to rest any major controversy concerning cost effectiveness [10]. In fact by the 1990s almost all NICUs in the high income countries had achieved ≥ 90% survival of babies with a birth weight of >1,000 g [10].

The world’s nations are divided in providing equity in newborn care [16] and the global picture of newborn care and outcomes presents an unfortunate contrast on both sides of the divide between high income countries *versus* low income countries [16–19]. The scenario and challenges of neonatal survival are unique in low income countries burdened by very high neonatal mortality [16] and socioeconomic and political constraints [17]. Worldwide some 12.9 million babies are born preterm each year; of which 1.12 million die each year (28% of 4 million global neonatal deaths; 98% of which occur in low income countries) [18]. The very high cost and highly technology dependent care of extremely premature babies (<28 weeks and <1,000 g at birth) is neither a viable nor a sustainable option for low income countries. Therefore UNICEF’s Annual Global Child Survival Reports exclude babies <1,000 g from the mortality data published for low income countries [19]. These countries might be better off investing their limited resources in the care of full term and bigger preterm babies, the majority of which die of potentially preventable diseases [16]. Indeed some developing countries e.g., Sri Lanka, Indonesia, Moldova, Nicaragua, Vietnam and Honduras have, within their limited resources and without resorting to very expensive high tech intensive care facilities, successfully reduced their neonatal mortality rates [17].

According to the March of Dimes 2009 Global Report on Preterm Births [18], there are huge gaps in the data with regards to preterm birth, prevalence, mortality, acute morbidity and long term impairment in low income countries. Such deficiencies in the available data makes the much needed systematic scaling up of neonatal care [20] in these countries an extremely difficult task. The present study aims to bridge this gap. We collected, analyzed and compared the gestational age specific mortality and morbidity data from the State of Qatar which has achieved excellent Neonatal mortality rates in recent years (4.37, 5.1, 4.4, and 4.0/1,000 births in 2006, 2007, 2008 and 2009 respectively) [19,21,22] with the data from the developed countries. In addition, the current study also explored the feasibility of facility based cost effective options for saving babies at the lowest limits of viability (28^+1^ to 32^+0^ weeks) in low income countries.

### 1.1. Aim of the Study

The present study analyzed the gestational age specific mortality and morbidity outcome of 28^+1^ to 32^+0^ weeks gestation babies in the State of Qatar over a period of five years (2002–2006), and compared the outcomes with recent data published in the well established international neonatal databases from some high income countries (VON [23] and NICHD [24]) and recent studies published from UK [1], France [2] and Europe [3,4]. The six specific objectives of our study were to:

- Develop a Performa, using the international databases (VON and NICHD) as a template, in order to collect the mortality and pre discharge in hospital morbidity of 28^+1^ to 32^+0^ weeks gestation babies in the State of Qatar based on the individual patient medical records;
- Retrieve data on gestational age specific mortality and morbidity outcome of 28^+1^ to 32^+0^ weeks gestation babies in the State of Qatar for the period (2002–2006); (N = 597 during the study period);
- Double check the validity of the data collected via the Performa using parallel databases in our institution (Hamad Medical Corporation);
- Appraise the Qatari mortality and pre discharge in hospital morbidity data and compare the outcomes with similar data from VON, USA (NICHD), UK, France and Europe;
- Analyze the characteristics of respiratory support needed at each successive gestational week among our sample (28^+1^ to 32^+0^ weeks) and compare it with data from Vermont Oxford Network (2007) report; and,
- Contribute towards finding evidence based cost effective neonatal care options for resource restricted developing countries which are struggling to reduce their burden of neonatal mortality.

## 2. Methods

### 2.1. Procedures and Sample

This retrospective analytic study was approved by the Institutional Research Board of Hamad Medical Corporation (protocol No. 7004/07), and undertaken in the Neonatal Intensive Care Unit Women’s Hospital in Doha (the only tertiary care maternity and neonatal unit in the State of Qatar). The Hospital was accredited in 2006 and re-accredited in 2009 by the Joint Commission International (JCI) USA for its Standards and Quality of Care. About 99% of deliveries in Qatar take place in this hospital. All inborn and out born babies delivered ≤32 week gestation in the State are admitted to the Women’s Hospital. Therefore the data with regards to ≤32 week gestation babies from our study not only accurately represents Qatar’s national data, for all practical purposes, it is equivalent to a population-based study.

### 2.2. Performa and Data Collection

A standard Performa based on established international neonatal databases like VON and NICHD as well as some recently published preterm mortality and morbidity outcomes data from the high income countries [2–4,23,24] was developed by the authors. Using the standard Performa, data was collected by two of the authors (H.P, K.P.N) from individual patient medical records using anonymous identification numbers. Due to the retrospective medical records based nature of the study, individual patient consent was not required by the Institutional Research Board. The Performa comprised data items that included a range of base line characteristics of antenatal care (Table 1); the use of antenatal steroids, mode of delivery, condition at birth, and respiratory management in the neonatal period (Table 2); mortality (Table 3); and, in hospital pre discharge morbidity in the survivors (Table 4). A Performa was completed for each of all 28^+1^ to 32^+0^ weeks gestation babies (N = 597) admitted during the study period (1st January 2002–31st December 2006). Twenty seven individual patient medical records (4.5%) were incomplete. This deficiency was completed from other parallel data bases (e.g., admissions, discharge and death registers of the neonatal unit). These parallel information sources were also used to double check the validity of the rest of the data.

### 2.3. Outcome Measures

We employed three major categories of outcome measures: (1) 28^+1^ to 32^+0^ weeks gestation birth rates; (2) in hospital pre-discharge mortality (total and gestational age specific); and, (3) in hospital pre-discharge morbidity (CLD, NEC, Symptomatic PDA, IVH Grade III and IV, Cystic PVL, ROP ≥ Stage 3). Chronic Lung Disease (CLD) was defined as supplemental oxygen dependence or ventilation including CPAP at 36 weeks corrected post-menstrual age (PMA). Neurologic morbidity (IVH and PVL) were defined using the Papile Classification [25]. We also analyzed the characteristics of respiratory support (e.g., MV, surfactant therapy, CPAP therapy and total days on respiratory support) needed at each successive gestational week among our sample (28^+1^ to 32^+0^ weeks). The comparative data for Vermont Oxford Network (VON) was ascertained from its 2007 report available in CD format and on its web site (www.vtoxford.org) [23]. VON has two sections in its database:

1. Low birth weight database (babies 501–1,500 grams at birth)
2. Expanded database (babies of all birth weight and gestational ages)

The expanded database classifies babies both according to the birth weight categories and gestational age categories. The gestational age category tables 19–32 of Expanded database in VON’s 2007 report presents data according to the following gestational age categories irrespective of their birth weight; <27, 27–29, 30–32, 33–36, 37–41 and >41 weeks. We used the 30–32 weeks group from expanded database gestational age categories which is the best nearest to our data (28^+1^ to 32^+0^). VON’s 30–32 weeks group in the expanded database includes babies of all birth weights (<1,500 g and >1,500 g). This makes the comparative groups very similar to ours. In VON database the outcomes are described as percentage (%) with 1st and 3rd network quartile without giving the actual numbers (*n*) in any category. VON gives the total and group specific number (N) of babies in the beginning of each table. We calculated the *n* for each category of VON tables using the 19–32% and the total numbers (N) given in the beginning of each table.

### 2.4. Statistical Analysis

The net based statistical package Vassar stat was employed for data analysis. The chi square (χ^2^) test was used to compare the Qatar data with that of VON and other high income countries. The significance level was set at *p* < 0.05.

## 3. Results

A total of 64,689 live births were recorded during the study period with 597 of these babies being babies between 28^+1^ to 32^+0^ weeks gestation, thus giving a prematurity rate of 9.23 per 1,000 total births for the target gestational age category during the study period. Our 28^+1^ to 32^+0^ weeks gestation prematurity rate was less than the rates for the same gestation group in the UK (*p* < 0.0001). Table 1 highlights the patient characteristics of our sample in comparison to VON, which is an international database of more than 800 Neonatal ICU’s; more than 95% of which are located in North America, Western Europe and Australia. 86.44% of our babies had birth weights between 1,001 and 2,000 g and only 3.9% were SGA as compared to 12% in VON database (*p* < 0.0001). Singleton babies comprised 81.91% of the sample. The gender distribution was equal, and almost 45% of the babies were born to native Qatari families and 55% to expatriate families living and working in Qatar. This is in line with the general distribution of population in Qatar [25]. The rate of antenatal steroid administration in our sample (86.1%) was better (p < 0.0001) than VON (73%). The 28^+1^ to 32^+0^ weeks mortality rate in our sample was 65.3/1,000, which was higher (p < 0.0001) than VON (29.9/1,000). 35.89% of deaths in our cohort were Early Neonatal Deaths (0–7 days of life) and 64.11% Late Neonatal Deaths (8–28 days of life). Lethal Congenital and Chromosomal Anomalies accounted for 12 deaths (30.77%); Trisomy 18 (four cases), Trisomy 13 (one case), multiple congenital anomalies (one case), and complex congenital heart disease (four cases including two with Down’s syndrome).

Table 2 shows the characteristics of the respiratory support required by the 28^+1^ to 32^+0^ weeks gestation babies of our cohort during their stay in the NICU. Slightly more than one third of our babies (37.52%, n = 224) did not require any respiratory support during their entire NICU stay. Of the remaining 373 babies (62.48%) who required respiratory support, 186 (31.3% of total and 50% of babies requiring respiratory support) needed CPAP alone. One hundred and eighty seven (31.3% of total and 50% of babies requiring respiratory support) needed MV. Among the babies requiring MV, 83 (13.9% of total babies and 44.4% of babies requiring MV) needed post extubation CPAP support. Surfactant therapy was required only by one third (32.16%) of total babies. The need for respiratory support decreased (*p* < 0.001) with increasing gestational age (MV decreased from 88.71% at 29 weeks to 13.26% at 32 weeks; CPAP decreased from 66.13% at 29 weeks to 31.44% at 32 weeks; and surfactant replacement decreased from 88.71% at 29 weeks to 15.53% at 32 weeks). Most of the respiratory support (80.12% MV, 96.28% CPAP) was required for short duration (<96 hours).

Table 3 depicts the total and gestational age specific mortality and survival rates with a comparison with recent data from UK [1]. Qatar’s average Neonatal Mortality Rates for the study period 6.34/1,000 and 28^+1^ to 32^+0^ weeks gestation mortality rate was 65.33/1,000. Qatar’s 28^+1^ to 32^+0^ weeks gestation survival rate of 93.47% was significantly less (*p* < 0.0001) than the same gestational age survival rate in UK during 2006 [1]. Within our own cohort the gestational age specific survival rate increased significantly (*p* < 0.001) with every single week increase in gestational age (from 85.49% at 29 weeks to 96.66% at 32 weeks).

Tables 4 illustrates the total and gestational age specific in hospital pre discharge morbidity of 28^+1^ to 32^+0^ weeks gestation babies in Qatar with comparative figures for 30–32 weeks gestation babies from the expanded data base in VON 2007 report [23].

Our incidence of CLD and symptomatic PDA was significantly less (*p* < 0.0001), while the incidences of NEC, IVH grade III, IVH grade IV and PVL were similar to VON. On the other hand, our incidence of ROP was significantly higher (*p* < 0.0001) than VON. Within our cohort the incidence of symptomatic PDA, NEC and ROP (≥Stage 3) decreased with every single week increase in gestational age (*p* < 0.05).

We constructed Table 5 using a subanalysis of our data restricted to babies with birth weight 1,001–1,500 g (n = 282). This included 205 babies from our study, as shown in Table 1, plus 77 babies who were <28 weeks gestation from another yet unpublished study conducted for the same time period (2002–2006) by our group on extremely low birth weight babies. This subanalysis made it more rational for us to compare our pre-discharge in hospital morbidity outcome with studies based on birth weight instead of gestational age. Table 5 depicts comparative data for 1,001–1,500 g babies between Qatar and recent studies from the USA (NICHD) [24], Italy (Trento study) [30] and UAE [31]. The incidence of pre-discharge respiratory and neurologic morbidity was very low among our cohort as compared to NICHD, but comparable to the Trento and UAE studies. Severe ROP (≥Stage 3) was high (5.69%) in our study as compared to all the other groups.

## 4. Discussion

Progress in neonatology is generally portrayed as inexorable: doing better and better with smaller and smaller [10]. However, this represents only one side of the challenge, reflecting the outcome of only 1% of global births which take place in high income countries. Of the 130 million babies born every year worldwide, 12.9 million are born preterm. Four million babies die in the first month of life; and 99% of these deaths occur in low and middle income countries due to causes which are largely preventable [16]. Preterm mortality accounts for 28% of global neonatal deaths [16]; (about 1.12 million deaths per year) [18]. This could possibly even be an under estimation because a large number of preterm births in low income countries take place in the community and are never reported [16]. Hence, low income countries are caught between the dilemma of limited human and economic resources and the reports of increasingly intact survival of very preterm babies particularly those born at >28 weeks gestation and >1,000 g at birth [10–14]. The rapid and widespread use of information technology has raised parental expectations all over the world. The unfortunate fact is that the resources available are limited, particularly in developing countries, and rationing is imperative. Therefore where and how to invest in neonatal care in low income countries remains a very important question [27].

Among the low income countries, Sri Lanka stands out for having achieved a significant decline in its neonatal mortality rates by sustained investment in primary health care facilities and without establishing many high tech expensive neonatal intensive care units [17]. However, any further reduction in neonatal mortality in low income countries, particularly among the preterm infants, will need some facility-based interventions. Some low cost interventions like regular use of antenatal steroids for preterm labor and the increasing use of non invasive respiratory support in the neonatal units has contributed significantly to the survival of extremely preterm babies in high income countries. The application of these low cost interventions may increase the intact survival of preterm babies at the limit of viability in the low income countries (28^+1^ to 32^+0^ weeks gestation) at an affordable cost. In order to explore this proposition, we carried out this analytic and comparative study of the outcome of 28^+1^ to 32^+0^ weeks gestation babies in the State of Qatar. The selection of Qatar is a model of study was based on two rationale: first, the Perinatal and Neonatal survival rates in Qatar have significantly improved over the last thirty years to the extent that its current rates are comparable to many high income countries [19,21,22]; and second, the population of Qatar displays great diversity in its genetic, cultural and social backgrounds. Almost 60% of the resident families are economic migrants, mostly from Middle East, South and South East Asia [26]. Since the State of Qatar provides equal access to health care for its residents, both local and expatriate, the outcomes are even among a population with diverse origins [26]. In other words if a similar level of care is provided in the native countries of expatriate residents, the outcomes would be equally good.

We conducted the current study with six very clear objectives described in the methodology section. The first three objectives were successfully met by developing a standard Performa based on international benchmarks [23,24] followed by its testing and validation using parallel data bases in our unit.

To meet the fourth objective of our study, we tabulated the characteristics of our patient population (Table 1) in comparison to similar characteristics employed in VON [23]. There was no difference in the sex distribution and plurality between our data and VON. The number of babies with intrauterine growth retardation (SGA) was very high (*p* < 0.0001) in VON (12%) as compared to Qatar (3.9%). The case (*p* < 0.0001) for babies delivered by caesarean section (67% *vs.* 54.27%) was similar. Intrauterine growth retardation provides a respiratory advantage in the immediate postnatal period because the incidence and severity of surfactant deficiency lung disease is less in Small for Gestational Age (SGA) babies which comprise a significant proportion of the births in the low income countries. According to our study, the birth rate of 28^+1^ to 32^+0^ weeks gestation babies in Qatar is 9.23/1,000 which is significantly less (*p* < 0.0001) than the rate of similar gestation babies from UK (11.6/1,000) [1], but significantly higher (*p* < 0.0001) as compared to the rate of 7.6/1,000 in France [3,4]. The assisted reproductive unit in Qatar was established in the mid 1990s. Since then the number of preterm as well as multiple births have gradually increased in the State. Approximately 97% of babies in our cohort were >1,000 g at birth; thus making it feasible to compare with some reports based on birth weight rather than gestational age.

We assessed our data (Tables 2, 3 and 4) for the three outcome measures (mortality, morbidity and requirements for respiratory support in the immediate postnatal period) and compared the outcomes with similar data from, UK [1], Europe [2], France [3,4], USA [24], Italy [30] and UAE [31].

Mortality: Qatar’s average NMR for the study period (2002–2006) was 6.34/1000, which was significantly higher (*p* < 0.001) than UK’s NMR in 2006 (3.4/1,000) [1]. However, year by year, Qatar’s NMR decreased significantly from 7.49/1,000 in 2002 and 8.49/1,000 in 2003 to 4.37/1,000 in 2006. Qatar’s 2006 NMR was comparable to UK’s 2006 NMR (*p* = 0.08). Qatar’s average survival rate of 28^+1^ to 32^+0^ weeks gestation babies during the study period (2002 to 2006) was 93.47%, which is very close to the rate in Europe (94.8%) [2] and France (95.36%) [3,4] but significantly less (p < 0.0001) than the 2006 rate in the UK (97.6%) [1]. Lethal congenital and chromosomal anomalies were the leading cause (31%) of death in our Qatari cohort. This might not be very surprising, as according to the March of Dimes Birth Defects Foundation Report published in 2006 [28], Arab countries have the highest incidence of birth defects in the world. The incidence of birth defects in Qatar is 73.4 per one thousand live births [28] which is very similar to other Arabian Gulf countries. The overall consanguinity rate in the Arab world ranges between 40 and 70% while Qatar’s consanguinity rate is (54%) [29]. At the same time, Qatar has a very low elective antenatal termination rate due to socio cultural and religious reasons. Collectively these factors have resulted in a very high birth defects rate in Qatar. Among European countries the rate of elective terminations for congenital anomalies varies from 0.5% in Poland to 14.6% in Italy and 17.6% in France [3]. An improved antenatal termination rate may help in reducing neonatal deaths due to lethal congenital and chromosomal anomalies in Qatar. Qatar’s 28^+1^ to 32^+0^ weeks survival rate was very close to that of Europe (94.8%) [2] and France (95.3%) [3,4]. The French study [3,4] excluded congenital anomalies from outcome data. A head to head analysis between Qatar and France, after excluding congenital anomalies in both the data sets, reveals a better outcome of 28^+1^ to 32^+0^ weeks gestation babies in Qatar. The survival rate of 28^+1^ to 32^+0^ weeks gestation babies in our study increased (*p* < 0.001) with increasing gestational age (from 85.94% at 29 weeks to 96.66% at 32 weeks); a trend similar to the patterns witnessed in the UK [1] and France [3,4].

Morbidity: The incidence of in hospital pre discharge morbidity (Table 4) of our cohort was better than VON [23] (symptomatic PDA 3.35% (*p* < 0.0001), NEC 2.51% (*p* = 0.057), IVH Grade III 0.84% (*p* = 0.086), Grade IV 0.5% (*p* = 0.032) and cystic PVL 0.5% (*p* = 0.032)). The incidence of CLD was significantly higher (*p* < 0.0001) in VON as compared to Qatar (10% *versus* 2.68%). According to the 2007 report of VON [23], 40% of babies born at gestational age between 30 and 32 weeks required MV, 60.57% required CPAP, while 39% were given surfactant. It is possible that the high rate of use of antenatal steroids in Qatari cohort (86.1% *vs.* 73% in VON *p* < 0.0001) and the fact that 69% of babies in qatari cohort required either no respiratory support or only short term CPAP may have contributed toward a significantly low incidence of CLD among 28^+1^ to 32^+0^ weeks gestation babies Qatar. Qatar’s higher mortality gestational age specific mortality rates during the study period may have lowered the rates of morbidities. In this study we had also analyzed the week wise mortality for each individual year from 2002 to 2006. The gestational age specific mortality decreased significantly in each group between 2002 and 2006. Qatar’s 2006 gestational age specific mortality and morbidities remain comparable to most of the international benchmarks.

We undertook a sub analysis for birth weight specific (1,001 to 1,500 g) morbidity outcomes in our cohort and compared our findings (Table 5) with the recently published NICHD data [24], the Trento Study from Italy [30] and a study from the United Arab Emirates [31]. Our rate of CLD was better than NICHD [24], comparable to UAE [31] and more than Trento [30]. While our rates of IVH grade III, grade IV and PVL were similar to other studies, the rates of NEC and ROP (≥Stage 3) were much higher than these studies. This suggests the need for further research into our very low birth weight feeding practices and oxygen saturation monitoring policies. Although 20 (3.35%) babies in Qatari cohort had hemodynamically significant PDA, only four babies (0.67%) required surgical ligation. Similarly, 34 (5.69%) babies in the Qatari cohort had ROP (≥Stage 3) but only six babies (1.0%) required laser therapy. The incidence of ROP decreased with increasing gestational age (11.74% at 29 weeks to 2.65% at 32 weeks). This decrease is in agreement with the pattern and intensity of respiratory support and hence use of supplementary oxygen which decreased with increasing gestational age. A similar pattern was also observed in a recent study of low birth weight babies from UAE [31].

Patterns of Respiratory support: For the fifth objective, in addition to analyzing the patterns of respiratory support we also analyzed the implications of our findings for low income countries in terms of cost and feasibility. Respiratory support, the immediate postnatal requirement of most preterm babies, has significantly changed its pattern over the last decade with marked reduction in the requirement of mechanical ventilation [32]. The change, which is particularly marked in babies ≥ 29 weeks gestation [32], may be a blessing for low income countries because the number of care days required by the ventilated babies is a major expense in the NICU [10]. In our cohort of 28^+1^ to 32^+0^ weeks gestation babies in Qatar, slightly more than one third of the total babies (37.5%) did not require any respiratory support while another one third of the total babies (31.1%) required short term (<96 hours) non invasive respiratory support with CPAP. The number of babies requiring CPAP decreased sharply with increasing gestational age (*p* < 0.001) and increasing post natal age (*p* < 0.001). Of the babies who required CPAP in our cohort, 55% required CPAP for <24 hours while another 40% required CPAP between 24 and 96 hours of age. Over the last decade not only the knowledge and practice of using CPAP and its variants in NICUs has tremendously increased with significant reduction in the need of intubated respiratory support [32,33]; but also low cost CPAP modalities have been introduced for developing countries [34]. The recently published experience of using low cost Bubble-CPAP in Fiji is very encouraging [34]. These changing patterns of respiratory support and innovation in CPAP therapy makes it possible to save the majority of the 28^+1^ to 32^+0^ weeks gestation babies in low resource countries without restoring to high cost MV and surfactant replacement therapy. In our cohort only one third of total babies (31%) required short term (<96 hours) MV and 32% of the total babies required surfactant replacement therapy. Some of the babies in our cohort were intubated, given surfactant and extubated to CPAP (INSURE therapy); hence the number of babies requiring surfactant is more than the babies given MV. The requirement for MV, in our cohort, decreased sharply with increasing gestational age and postnatal age (p < 0.001). Antenatal steroids enhance the foetal “lung maturation” by increasing the surfactant production and release; thus reducing the need of respiratory support in the immediate postnatal period with improvement in survival of preterm infants [32]. Our very good rate of antenatal steroid administration (86.1% as compared to 73% in VON) and 100% surfactant replacement therapy in ventilated babies may have contributed significantly towards short term (<96 hours) gentle ventilation in the immediate postnatal periods as well as to the low incidence of CLD (2.68%) in our cohort.

Our data is stratified according to gestational age which is a better indicator of maturity, though most of the publications and some standard neonatal databases (e.g., NICHD) are stratified according to the birth weight [24]. VON data is stratified both according to the gestational age and birth weight [23]. Due to these different stratification methods, it was difficult for us to generate a head to head comparison with other studies including VON in which the gestational age stratification group (30–32 weeks) was more closer to ours (28^+1^ to 32^+0^ weeks). However, as shown in Tables 4 and 5, the general patterns of mortality and incidence of in hospital pre-discharge morbidities are similar between these data bases and our study. The recently published birth weight stratified data from Italy [30] and UAE [31] have shown a lower incidence of in hospital pre discharge morbidity as compared to VON. Our morbidity pattern was similar to the study from Italy [30] and UAE [31]. A recent review of NICHD data base has found a wide range of morbidity and mortality among network centres [24]. A similar variation was reported by the MOSAIC study data base from 10 different European regions [3,4]. This indicates that some best practices and currently available therapies are awaiting a formal discovery [24] which may help change the fate of millions of babies in the low income countries [35].

For the sixth objective, driven by Qatari data, we tried to find evidence on how and where to invest in neonatal care in low income countries to reduce the burden of four million total and 1.12 million preterm neonatal deaths; a major challenge faced by Perinatal health care planners [17,27]. A point to note is that major reductions in neonatal mortality can be achieved, within resource restricted environments, by sustained input at the community and primary health care level which must stay as the highest priority. Sri Lanka, Vietnam, Indonesia, Honduras, Moldova and Nicaragua are low income countries with stories of success [17] in achieving significant reduction in neonatal mortality rates in resource restricted settings. For low income countries, the lower limit of viability should stay at 28 weeks of gestation and 1,000 g birth weight as accepted by the UNICEF [19]. The experience from Qatar, to which our study is evidence, demonstrates that excellent preterm survival rates with minimum morbidity, comparable to high income countries are achievable. With limited resources, the facility-based services required for the care of preterm babies will have to be scaled up. Our study demonstrated that the vast majority of babies at the limit of viability for low income countries (28^+1^ to 32^+0^ weeks gestation) can be saved by using low cost techniques including extensive use of antenatal steroids and post natal CPAP therapy which, of course will remain complementary to the basic needs of temperature maintenance, nutrition including breast feeding, and prevention and management of infection [20,35].

## 5. Conclusions

The State of Qatar has achieved an excellent rate of intact survival of 28^+1^ to 32^+0^ weeks gestation babies which is comparable to that of many high income countries. Qatar’s data demonstrates that low cost techniques (antenatal steroids for preterm labor and post natal use of CPAP therapy), can potentially save the majority of 28^+1^ to 32^+0^ weeks gestation babies in low income countries with minimum in hospital pre discharge morbidity. The assessment of long term morbidities (at two years neurodevelopmental follow up) will be the true determinant of the ultimate outcome. Further, up to date studies in this area will be helpful for prioritizing health care investments in resource constrained countries.

## Figures and Tables

**Table 1.** Comparison of patient characteristics between Qatar and Vermont Oxford Network (VON).

**Characteristic**	**Qatari Sample *N* (%)**	VON*N (%)	*P* –Value**
**Birth Weight** (grams)			
<1,000	19 (3.18)	—	
1,001–1,500	205 (34.34)		
1,501–2,000	311 (52.1)		
>2,000	62 10. 38)		

**Gender**			
Male	295 (49.41)	6,222 (54)	
Female	302 (50. 59)	5,301(46)	

**Ethnicity**			
Qatari	269 (45.06)	White 6,914(60)	
Non Qatari	328 (54. 94)	NonWhite 4,609(40)	

**Plurality**			
Singleton	489 (81. 91)	8,873 (77)	
Twins (39 × 2)	78 (13.06)	1,613 (14)	
Triplets (10 × 3)	30 (5. 03)	1,037(9)	

**Intrauterine Growth**			
SGA	23(3. 9)	1,383 (12)	<0.0001

**Antenatal Steroids**			
Yes	514 (86.1)	8,412 (73)	<0.0001

**Caesarean Section**			
Yes	324 (54. 27)	7,720 (67)	<0.0001

**Respiratory Support**			
Yes	373 (62.48)	—	

**Surfactant Therapy**			
Yes	192 (32.16)	4,312 (37.4)	

**Symptomatic PDA**			
Total	20 (3.35)	1,498 (13)	
Medical Treatment	16 (2.68)	461 (4)	
Surgical Treatment	4 (0.67)	115 (1)	

**ROP ≥ Stage 3**			
Total	34 (5.69)	115 (1)	
Surgical Treatment	6 (1.0)	0 (0)	

**Mortality Rate/1000**			
Total	39 (65.3/1,000)	333 (29.9/1,000)	<0.0001
Early (<7d)	14 (23.45/1,000)		
As % of Total Mortality	35. 89%		
Late (>7d)	25 (41.87/1,000)		
As % of Total Mortality	64.11%		

**Causes of Mortality**			
Lethal Congenital & Chromosomal Anomalies	12 (30.77)		
Sepsis	11 (28.20)	—	
Severe Birth Asphyxia	6 (15. 30)		
NEC	4 (10. 25)		
Pulmonary Hemorrhage	3 (7.69)		
Hydrops / Congenital Infection	3 (7.69)		

Total Babies: Qatari Cohort (29–32 week) = 597, VON (30–32 weeks) = 11,523;

* Data represents 30–32 weeks gestation group from Expanded data base VON 2007 report [23]

** Chi Square test used to calculate *p-*value.

**Table 2.** Characteristics of Respiratory Support in Qatar for 28^+1^ to 32^+0^ weeks gestation babies.

**GA Weeks**	**N**	**RS *n* (%)**	**Surf *n* (%)**	**MV *n* (%)**	**CPAP *n* (%)**
29	62	58 (93.55)	55 (88.71)	55 (88.71)	41 (66.13)
30	116	95 (82.00)	42 (36.21)	48 (41.38)	63 (54.31)
31	155	110 (71.0)	49 (31.61)	49 (31.61)	82 (52.9)
32	264	110 (41.67)	41 (15.53)	35 (13. 26)	83 (31.44)
**Total**	597	373 (62.48)	192 (32.16)	187 (31.32)	269 (45.06)*
*p* Value**		<0.001	<0.001	<0.001	<0.001

GA: gestational age; RS: Respiratory Support; Surf: Surfactant therapy; MV: Mechanical Ventilation; CPAP: Continuous Positive Airway Pressure;

* CPAP alone: 186 (31.1%) CPAP following MV: 83 (13.9% of total babies and 44.4% of babies requiring MV)

** p-value calculated using the Chi Square test (comparing decreasing respiratory support with increasing GA).

**Table 3.** Comparative analysis of total and gestational age specific mortality and survival rates: Qatar *versus* high income countries.

**Parameter**	**Qatar (2002–6)**	**UK (2006)^a^**	***P* Value**
Total Births	64,689	669,465	

Neonatal Mortality^b^	410 (6.34/1,000)	2,305(3.44/1,000)	<0.0001

Neonatal Survival (%)	99.4	99. 6	

Premature Births	597 (29–32 weeks)	7,770* (29–32 weeks)	

Prematurity Rate (/1,000)	9.23 (29–32 weeks)	11.6* (29–32 weeks)	<0.0001

29–32 Weeks gestation			
Total	597	7,770*	
Mortality *n* (/1,000)	39(65.33)	189(24.32)	<0.0001
Survival (%)	93.47	97. 6	

29 Weeks gestation			
Total	62	1,243	
Mortality *n* (/1,000)	9(145.16)	61(49.07)	<0.005
Survival (%)	85.49	95.1	

30 Weeks gestation			
Total	116	1,569	
Mortality *n* (/1,000)	15(129. 31)	52(33.14)	<0.0001
Survival (%)	87.07	96.7	

31 Weeks gestation			
Total	155	2,066	
Mortality *n* (/1,000)	6(38.71)	43(20.81)	=0.24
Survival (%)	96.13	97. 9	

32 Weeks gestation			
Total	264	2,892	
Mortality *n* (/1,000)	9(34. 09)	33(11.4)	=0.005
Survival (%)	96. 66	98. 9	

a UK Office of National Statistics Bulletin Published May 28, 2009 [1]; (*calculated from the reference)

b Qatar’s average NMR for 2002–2006. Qatar’s NMR for 2006 was 4.37/1,000.

**Table 4.** Total and gestational age specific in hospital pre discharge morbidity in Qatar compared with VON data (30–32 weeks).

**Gestation**	**N**	**CLD***n* (%)	**PDA***n* (%)	**NEC***n* (%)	**IVH G-III***n* (%)	**IVH G-IV***n* (%)	**Cystic PVL***n* (%)	**ROP ≥Stage 3***n* (%)
29 Weeks	62	5 (8.1)	5(8.06)	2(3.22)	3(4.81)	1 (1.61)	2(3.2)	11(17.74)
30 Weeks	116	7 (6.0)	12(10.34)	7(6.03)	1 (0.86)	1 (0.86)	1(0.86)	8(6.89)
31 Weeks	155	3 (1.94)	3(1.93)	4(2.58)	1 (0.65)	0	0	8(5.16)
32 Weeks	264	1 (0.38)	0	2(0.75)	0	1(0.38)	0	7(2.65)
**Total**	597	16 (2.68)	20(3.35)	15(2.51)	5(0.84)	3(0.5)	3(0.5)	34(5. 69)
VON*	1,1523	1,498(13%)	1,498(13%)	346(3%)	115(1%)	115(1%)	115(1%)	115(1%)
*p*-value**		<0.0001	<0.0001	=0.57	=0.86	=0.32	=0.32	<0.0001

CLD: Chronic Lung Disease; PDA: Patent Ductus Arteriosus; NEC: Necrotizing Enterocolitis; IVH: Intraventricular Hemorrhage; G-III: Grade III; G-IV: Grade IV; PVL Periventricular Leukomalacia; ROP: Retinopathy of Prematurity;

* VON report 2007 Data for 30–32 weeks gestation [23];

** Chi Square was used to calculate *p* value.

**Table 5.** Comparative Analysis of in hospital pre discharge Morbidity Rates for 1,001–1,500g birth weight babies: Qatar *versus* NICHD, Trento and UAE.

**Study**	**N**	**CLD***n* (%)	**PDA***n* (%)	**NEC***n* (%)	**IVH G-3***n* (%)	**IVH G-4***n* (%)	**Cystic PVL***n* (%)	**ROP ≥Stage 3***n* (%)
NICHD^24^	9,841	* (10)	* (18)	—	* (5)	* (2)	* (1.5)	—
Trento 30	166	2 (1.2)	16 (9. 6)	1 (0.6)	2 (1.2)	3 (1.8)	4 (2.4)	0 (0)
UAE^31^	110	6 (5.5)	—	5 (4.5)	1 (1)	0 (0)	1 (1.1)	0 (0)
QATAR**	282	15 (5. 3)	20 (7.1)	10 (3.5)	5 (1.8)	4 (1.4)	3 (1.1)	7 (2.5)

NICHD: National Institute of Child Health and Human Development; UAE: United Arab Emirates;

* Absolute numbers not provided in the NICHD Study; [24];

** Sub analysis of our data restricted to babies with BW 1,001–1,500 g (205 babies from our study plus 77 babies from < 28 weeks gestation cohort from another study undertaken by our group for the same time period 2002–2006).

## Abbreviations:

CLD: Chronic Lung Disease
NEC: Necrotizing Enterocolitis
PDA: Patent Ductus Arteriosus
IVH: Intra Ventricular Hemorrhage
PVL: Periventricular Leukomalacia
ROP: Retinopathy of Prematurity
MV: Mechanical Ventilation
CPAP: Continuous Positive Airway Pressure
VON: Vermont Oxford Network
NICHD: National Institute of Child Health and Human Development
